# Indoleamine 2,3-Dioxygenase 1 (Ido1) Is Involved in the Control of Mouse Caput Epididymis Immune Environment

**DOI:** 10.1371/journal.pone.0066494

**Published:** 2013-06-20

**Authors:** Aicha Jrad-Lamine, Joelle Henry-Berger, Christelle Damon-Soubeyrand, Fabrice Saez, Ayhan Kocer, Laurent Janny, Hanae Pons-Rejraji, David H. Munn, Andrew L. Mellor, Najoua Gharbi, Rémi Cadet, Rachel Guiton, Robert J. Aitken, Joël R. Drevet

**Affiliations:** 1 GReD laboratory, CNRS UMR 6293 - INSERM U1103 - Clermont Université, Aubière, France; 2 CHU Estaing, Assistance Médicale à la Procréation, CECOS, Clermont-Ferrand, France; 3 Immunotherapy Center and Department of Pediatrics, Medical College of Georgia, Augusta, Georgia, United States of America; 4 Immunotherapy Center and Department of Medicine, Medical College of Georgia, Augusta, Georgia, United States of America; 5 Faculté des Sciences de Tunis, El Manar, Département de Biologie, Mutuelleville, Tunis, Tunisia; 6 Priority Research Centre in Reproductive Science, School of Environmental and Life Sciences, University of Newcastle, Callaghan, Australia; INSERM-Université Paris-Sud, France

## Abstract

The epididymis maintains a state of immune tolerance towards spermatozoa while also protecting them and itself against infection and acute inflammation. The immunosuppressive enzyme indoleamine 2,3-dioxygenase 1 (Ido1) participates in this delicate local equilibrium. Using the mouse *Ido1^−/−^* model, we show here that the absence of IDO1 expression leads in the epididymis but not in serum to (1) an increase in the inflammatory state as evidenced by changes in the content of cytokines and chemokines, (2) the engagement of a Th1-driven inflammatory response as evidenced by changes in the Th17/Treg as well as Th1/Th2 equilibria, as well as (3) differences in the content of lipid intermediates classically involved in inflammation. Despite this more pronounced inflammatory state, *Ido1^−/−^* animals succeed in preserving the local epididymal immune situation due to the activation of compensatory mechanisms that are discussed.

## Introduction

Tryptophan (TRP) metabolism in mammals *via* indoleamine 2,3-dioxygenase (IDO) has received considerable attention in recent years because of its role in modulating both innate and adaptative immune responses (reviewed in: [1], [2]). First described as an effector of innate immunity, IDO and its associated kynurenine (KYN) metabolites are now mostly known for modulating the adaptative immune response, in particular T-cell mediated responses [1]–[3]. In most tissues, high KYN production directly reflects the anti-inflammatory and immunosuppressive enzyme activity of IDO and thus, the inflammatory and tolerogenic states of tissues.

In the mouse epididymis, IDO and the KYN pathway are constitutively activated [4]–[6]. In previous work we reported that epididymal IDO1 expression in the mouse is restricted to the caput region from segments 2 to 5 with a peak of expression in segments 3 to 4 [7]. Both principal and apical cells were shown to be reactive towards an anti-IDO1 antibody and the enzyme was shown to be cytosolic and absent from the epididymal luminal compartment [5], [7]. IDO1 is not the sole TRP-recycling enzyme found expressed in the mouse epididymis epithelium since TDO (tryptophane 2,3 dioxygenase) and INDOL (or IDO2, an homologous enzyme coming from a gene duplication event in mammals) were also shown to be expressed however at much lower levels [7]. We also demonstrated that IDO1 is the major contributor to KYN formation in the caput epididymis [7] since none of the other TRP-recycling enzymes compensated for the lack of IDO1 either at the transcription level or by their increased activities in a mouse model invalidated for IDO1, leading to a drastic drop in KYN content in IDO1-deficient epididymis extracts [7]. Kynurenine levels in mouse epididymal extracts were shown to be very significant and, amongst the various downstream products resulting from IDO1 activity we reported that L-kynurenine (KYNU), kynurenic acid (KYNA) and 3 hydroxykynurenine (3OHK) were by far the most abundant species following the caput expression of their corresponding generating enzymes in the kynurenine pathway [7]. In addition, we have shown that spermatozoa are capable of triggering *Ido1* expression upon entering the luminal compartment of the epididymis [7]. The constitutive expression of *Ido1* in the proximal adult mouse epididymis suggested that this region of the organ is permanently engaged in both anti-inflammatory and immunosuppressive actions needed to preserve spermatozoa, which are allogenic strangers to the male immune system, from auto-immune responses while maintaining an efficient anti-infection surveillance of the immune-privileged epididymal duct.

In the present report, we have analyzed in more depth the immune/inflammatory status of the caput epididymidis in both WT and IDO1-deficient mice in order to gain further insights into the role of IDO1 in controlling the balance between immunological responsiveness and tolerance.

## Materials and Methods

### Animals

The present study was approved by the Regional Ethic Committee in Animal Experimentation (CEMEA-Auvergne; Authorization CE2-04) and adhered to the current legislation on animal experimentation in France. Wild type and *IDO1^−/−^* BALB/c male mice [8] aged 6 months were used throughout the study unless otherwise indicated. Mice were housed under controlled environmental conditions (22°C, 12-h dark period), fed a basal diet (Global-diet, 2016S, Harlan, Gannat, France) *ad libitum*, and given free access to water. Mice were killed by cervical dislocation under CO_2_ anaesthesia. Proteins were extracted from liquid nitrogen-frozen epididymal tissues stored at −80°C until use. Briefly, tissues were homogenized in 20 mM HEPES, 0.42 M NaCl, 1.5 m MgCl_2_ M, 0.2 mM EDTA, 1% NP40, 1 mM phenylmethylsulfonyl fluoride, 0.1 mM Na_3_VO_4_, 0.1 mM NaF and complete 1X (Roche Diagnostics, Meylan, France). Blood samples were kept on ice in heparin-coated tubes, centrifuged 15 min at 1500 *g* at 4°C and then the plasma was recovered and kept at −80°C until use.

### Kynurenine, Tryptophan Determinations

TRP and KYN concentrations were measured by high performance liquid chromatography (HPLC) using 3-nitro-l-tyrosine as internal standard [9]. For separation, reversed-phase cartridges 55-mm LiChroCART RP18 column were used. TRP was detected by a fluorescence detector at an excitation wavelength of 285 nm and an emission wavelength of 365 nm. A Shimadzu (Marne la Vallée, France) SPD-6A UV-detector in flow stream series connection was used for detection of both KYN and nitrotyrosine at a wavelength of 360 nm. The elution buffer was a degassed potassium phosphate solution (0.015 M, pH 6.4) containing 5% methanol. Isocratic analyses were carried out at a flow-rate of 0.8 ml/min and a temperature of 25°C. Frozen serum and tissue specimens were thawed at room temperature, 200 µl of samples were diluted with 200 µl of potassium phosphate buffer (0.05 mol/l, pH 6.0) containing the internal calibrator 3-nitro-l-tyrosine (100 µmol/l). Protein was precipitated with 50 µl of trichloroacetic acid (2 M). The capped tubes with the precipitate were immediately vortex-mixed and centrifuged for 10 min at 13,000 *g*. One hundred and fifty µl of the supernatants were transferred into microvials and placed into the autosampling device. The external calibrator was prepared from freshly thawed stock solutions of TRP and KYN (1 mM in bidistilled water, stored at −20°C). Fifty µl of TRP, 10 µl of KYN, and 940 µl of albumin stock solution were mixed together. Two hundred µl aliquots of this external calibrator preparation were run through the entire analytic procedure in parallel with the biological specimens. Neither degradation of TRP nor production of KYN were observed in the calibration preparations, and our standards confirmed the stability of KYN and TRP as compared to protein-free standard preparations measured without any precipitation step.

### Measurement of Chemokine/Cytokine Levels

Commercially available mouse inflammation antibody arrays (RayBiotech Inc., Norcross, GA, USA) were used to detect chemokine and cytokine expressions. Plasma and caput epididymal protein extracts were treated as described by the supplier. Protein levels were estimated by densitometric analysis of the membranes. Each set of membranes contains positive and negative controls that were used for standardization and data are expressed as relative densitometric values. Chemokine/cytokine evaluations were performed on 3 biological replicates and 2 technical repeats were carried out in 3 distinct assays. For statistical analysis, ANOVA was performed with the factors “genetic background [WT vs KO]” and “assays” (Statgraphics Plus version 5.1). When the factor “genetic background” was significant, Fisher LSD tests (P<0.05) were used. The mean values and standard errors of mean values are shown on the figures.

### Fatty Acids and Sphingolipids Measurements

Total plasma lipids were extracted as described in [10]. The phospholipid fraction was separated by thin-layer chromatography on a silica gel plate (Merck, Darmstadt, Germany) and 1-stage mobile phase development, which consisted of the solvents hexane, ethylic ether and acetic acid in an 80∶20:2 (v/v) ratio. The plates were dried and sprayed with dichlorofluorescein to visualize cholesterol esters, phospholipids, triacylglycerols, and free fatty acid bands under ultraviolet light. The phospholipid band was scraped off into a separate test tube, and the fatty acids were converted into methyl esters. Fatty acid methyl esters were prepared and analyzed as previously described [11]. Ceramide and sphingomyelin analyses were performed at the lipidomic platform (INSERM IFR150 Metatoul Platform, Toulouse, France) as follows: cells were homogenized in methanol/EGTA 5 mM (2∶1 v/v) with a FAST-PREP apparatus (MP Biochmicals), and an aliquot was taken up for protein measurement. Total lipids were extracted in chloroform/methanol/5 mM EGTA (2.5∶2.5∶2.1, v/v/v) in the presence of the internal standard stigmasterol (18 mg) as described in [12]. Ceramide-NC15 (2 mg), was prepared according to [13]. The lipid phase was dried down under a nitrogen ﬂux, and lipid extract was submitted to a mild-alkaline treatment in 1 ml methanolic NaOH 0.6 N, followed by silylation in BSTFA (N,O-bis(trimethylsilyl)trifluoroacetamide ) containing 1% TMSCl (Chlorotrimethylsilane)/acetonitrile (1∶1, v/v) [13]. The mixture (5 ml) was directly analysed by gas-liquid chromatography on a 4890 Hewlett Packard (Palo Alto, CA, USA) system using a RESTEK RTX-50 fused silica capillary columns (30 m ×0.32 mm, 0.1 mm film thickness) [13]. Oven temperature was programmed from 195°C to 310°C at a rate of 3.5°C per min (for 12 min), and the carrier gas was hydrogen (0.5 bar). The injector and the detector were at 310°C and 340°C, respectively. SM and ceramide were separated on the basis of the number of carbons and the unsaturation of the amide-linked chain and quantified according to the internal standard. C16, C18, C22 and C24 SM or ceramide refer to the sum of the molecular species having this number of carbons in the lateral chain, independent of the unsaturation. Results are given as nmol per mg protein.

### Quantitative Reverse Transcriptase Polymerase Chain Reactions

Total RNAs were isolated with the NucleoSpin® RNA II kit (Macherey-Nagel, France) and were reverse transcribed by M-MLV Reverse Transcriptase (Promega Corp., France) according to the manufacturer’s instructions. Quantitative real time PCR assays were performed using a RealPlex thermocycler (Eppendorf). Two µl of diluted cDNA template (1/20) were amplified using MESA GREEN qPCR MasterMix Plus (Eurogentec, France) according to the manufacturer’s instructions. Primer sequences are given in Table 1 and amplification efficiencies are provided. To ensure no genomic DNA contamination primers were designed in distinct exons separated by at least 500 bp-introns, so that the genomic DNA cannot be amplified. A standard curve of amplification efficiency for each set of primers was generated with a serial dilution of plasmids containing DNA of targeted genes. Melting curve analysis was carried out to confirm the specificity of primers. For quantification of transcripts, the relative method was used to calculate mRNA relative level to *Cyclophilin B* standard which was chosen because it has a stable expression between the tissues and genotypes.

**Table 1:** Oligonucleotides used in the course of the study.

Gene product		Primer sequences (5 = >3′)	Insert length	Tm in °C	Efficiency
RorcNM_011281.2	Fw	AGCAGTGTAATGTGGCCTAC	179 bp	59°C	0.9886
	Rv	GCACTTCTGCATGTAGACTG			
FoxP3NM_001199348.1	Fw	CCCAGGAAAGACAGCAACCTT	90 bp	59°c	0.9778
	Rv	TTCTCACAACCAGGCCACTTG			
Cyclophilin bNM_011149.2	Fw	GGAGATGGCACAGGAGGAA	76 bp	55 to 63°C	0.9741
	Rv	GCCCGTAGTGCTTCAGCTT			
Gata-3NM_008891.3	Fw	ACGGAAGAGGTGGACGTACT	242 bp	58°C	0.9905
	Rv	GTGGTGGATGGACGTCTTG			
T-betNM_019507.2	Fw	GTTCCCATTCCTGTCCTTC	211 bp	60°C	09741
	Rv	CCTTGTTGTTGGTGAGCTT			
Cox1NM_008969.3	Fw	CATGGCTGGCCTAGAACTCACT	72 bp	63°C	0.9923
	Rv	AAGGCAGAGAGCAGTTGCATCT			
Cox2NM_011198	Fw	CCAGCACTTCACCCATCAGTT	52 bp	63°C	0.9868
	Rv	ACCCAGGTCCTCGCTTATGA			

### Western Blots

Proteins (40 µg) were separated by SDS-PAGE and transferred onto nitrocellulose membrane (Hybond ECL, GE Healthcare Biosciences, Piscataway, NJ). Blots were blocked with 10% low-fat dried milk/0.1% Tween 20/Tris Base Salt (TBS) and probed overnight at 4°C with anti-GAPDH (1/5000, Sigma-Aldrich) for loading controls and in parallel with various primary antibodies. For monitoring the activation of the STAT protein family, antibodies came from the STAT/Phospho-STAT antibody sampler kit (Cell Signaling, Ozyme, Saint-Quentin en Yvelines, France). For monitoring the activation of the Smad intracellular effectors, rabbit monoclonal anti-mouse Smad1, rabbit monoclonal anti-mouse Smad3, rabbit polyclonal anti-mouse Smad5 as well as their coreesponding rabbit monoclonal anti-mouse phospho-Smad1/5 and phospho-Smad3 (Cell Signaling, Ozyme, Saint-Quentin en Yvelines, France) were used. Each primary STAT (STAT1-3-5-6), Phospho-STAT (P-STAT1[Tyr701], P-STAT3[Ser727], P-STAT5[Tyr694] and P-STAT6[Tyr641]), Smad (Smad 1-3-5) and Phospho-Smad (P-Smad1/5 [Ser463/465] and P-Smad3 [Ser423/425]) antibody was used at a dilution of 1/1000 in 1X TBS-Tween 5%. The secondary antibody was a goat anti-rabbit horseradish peroxidase conjugate (1/5000, GE Healthcare, UK) that was detected using the ECL Western Blotting Detection kit on Hyperfilm™ (GE Healthcare). Densitometric analyses were carried out with “Quantity one” software (Bio-Rad, Marnes-la-Coquette, France).

### Fluorescence-Activated Cell Sorting Analyses

Caput epididymides were dissected and processed for the preparation of stroma-vascular fractions (SVF) as described in [14] Caspar-Bauguil *et al.* (2005). Briefly, tissues were digested in phosphate buffer saline (PBS) containing 2% bovine serum albumin (BSA) and 2 mg/ml collagenase (type II collagenase, Sigma-Aldrich, St. Quentin Fallavier, France) for 30 min at 37°C. Digested tissues were filtered through a 25 µm nylon membrane and tissue samples were centrifuged (600 *g* for 10 min) to generate SVFs. Cells were washed in PBS-5% FCS (fetal calf serum, Invitrogen, Cergy-Pontoise, France). Red cells were lysed in a buffer containing 155 mM ammonium chloride, 20 mM Tris, pH 7.6, for 5 min. Cells were then centrifuged (600g for 10 min) and re-suspended in PBS before counting on a Coulter counter (Beckman-Coulter, Roissy, France). Fluorescein (FITC)- or Phycoerythrin (PE) conjugated anti-cluster of differentiation 3 (CD3), CD4, CD8, CD19 antibodies were used (Miltenyi, France). Cells (around 2×10^5^/sample) were stained in PBS containing 5% mouse serum and incubated with conjugated anti-mouse monoclonal antibodies for 20 min at room temperature in the dark. Cells were washed in PBS and then analyzed on a fluorescence-activated cell sorter (FACS; FacsCalibur, Becton Dickinson Biosciences, France). Subpopulations of lymphocytes were identified by appropriate marker combinations and light scatter properties. Data acquisition was performed with Cell Quest software (Becton Dickinson = BD) while data analysis was done using BD Diva software.

### Statistical Analyses

*Kruskal-Wallis* and *Mann-Whitney* tests were performed with GraphPad Prism 5.02 software to determine the significance of differences between samples. *P* values ≤0.05 were regarded as significant.

## Results

**Figure 1:**
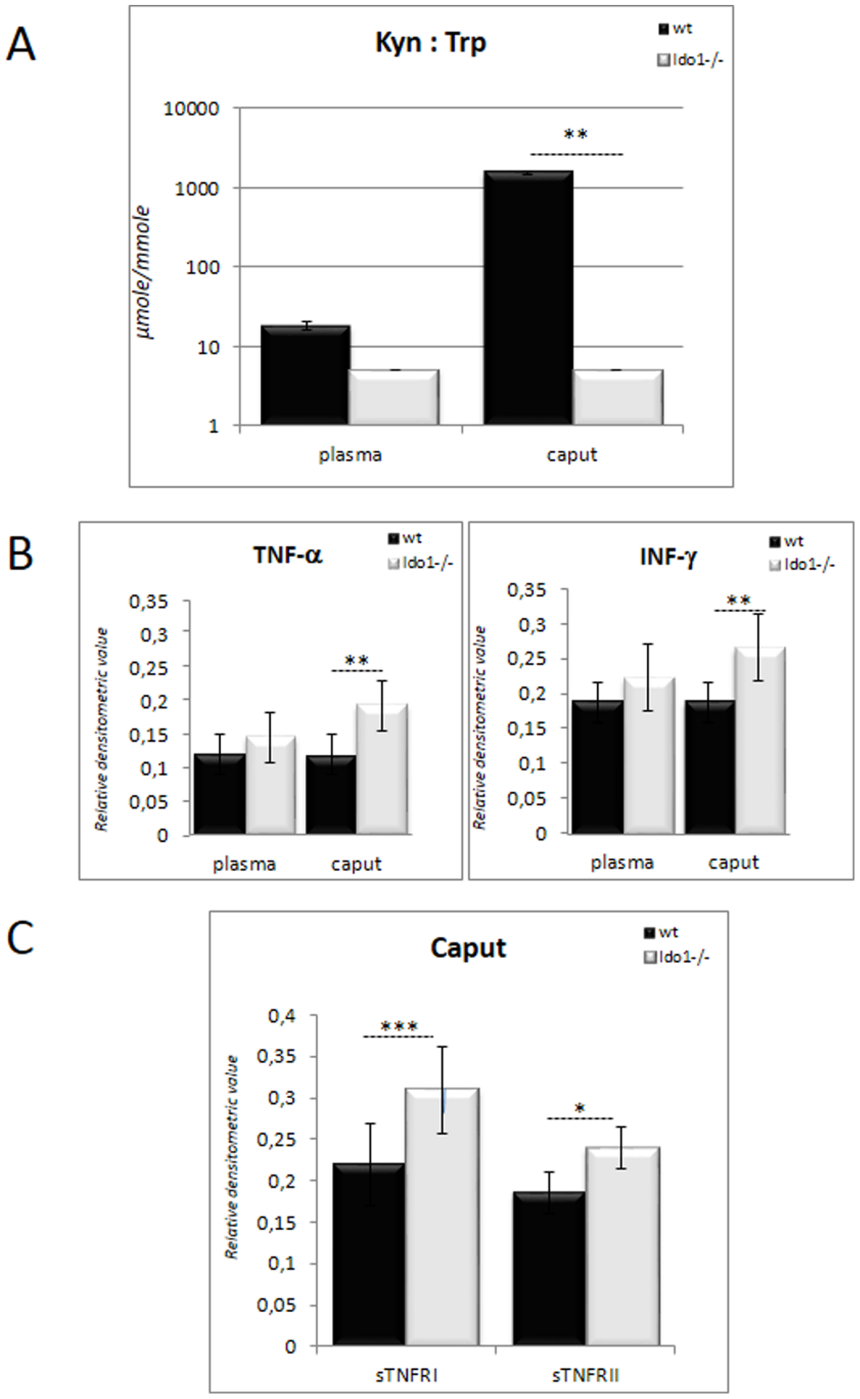
Analysis of the inflammatory status of the epididymis. **A:** KYN:TRP ratios in caput epididymis and blood plasma, respectively, of 6 month-old *wt* and *IDO1−/−* animals. The Y-axis is a semi logarithmic representation in µmole/mmole. **B:** Histograms show the levels of INF-γ and TNF-α in caput epididymidis extracts and plasma from 6 month-old *wt* and *IDO1−/−* animals. **C:** Histogram shows the levels of the soluble TNF receptors (sTnfRI and sTnfRII) in caput epididymidis extracts from 6 month-old *wt* and *IDO1−/−* animals. *P≤0.05, **P≤0.01, ***P≤0.001.

**Figure 2:**
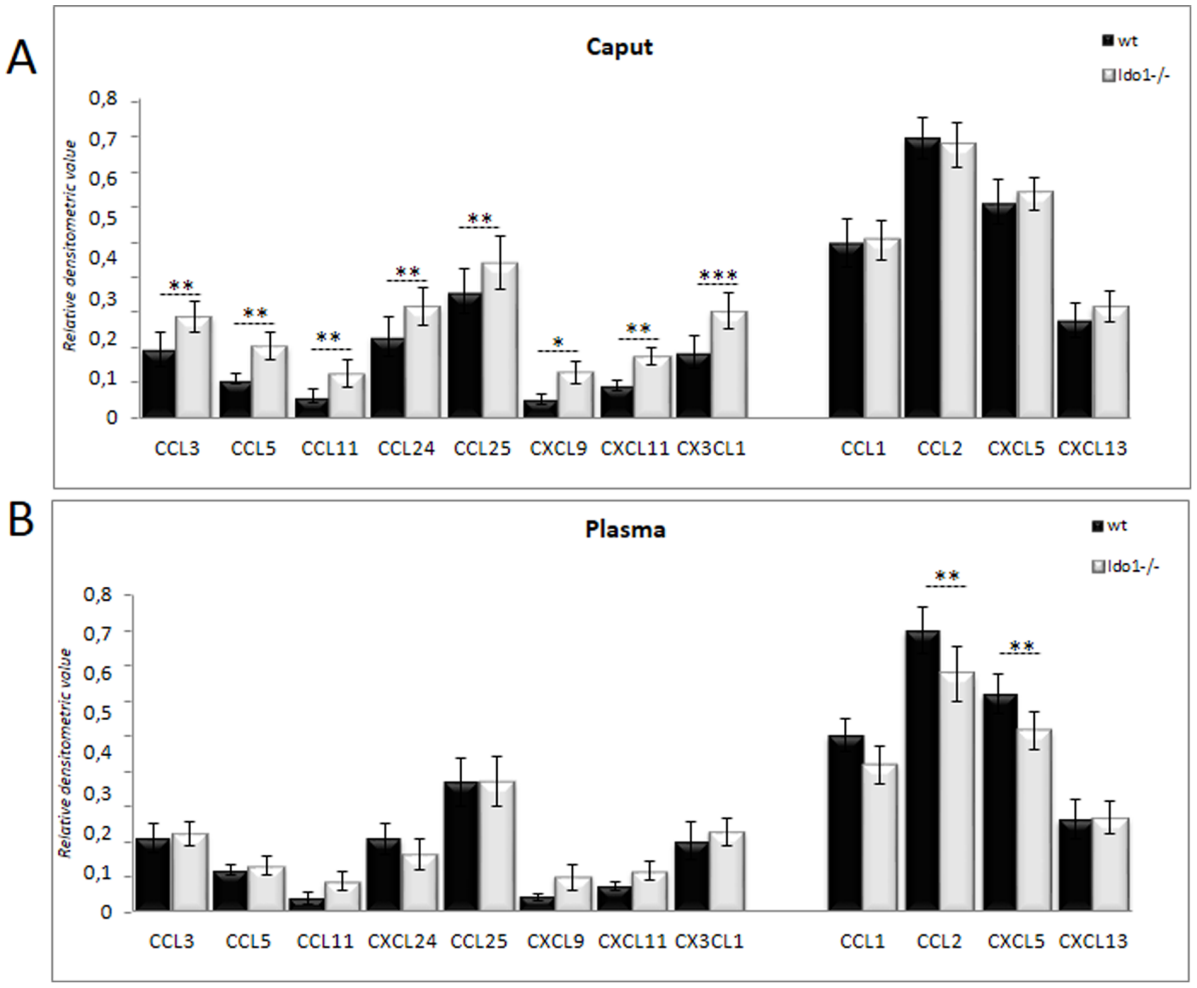
Chemokine profiles of the epididymis and plasma. Histograms show the levels of the inflammatory chemokines CCL1 (TCA-3, T-cell activation 3), CCL2 (MCP1, Monocyte chemoattractant protein 1), CCL3 (MIP-1α, Macrophage inflammatory protein 1), CCL5 (RANTES, regulated on activation normal T cell expressed and secreted), CCL11 (Eotaxin 1), CCL24 (Eotaxin 2), CCL25 (TECK, Thymus-Expressed Chemokine), CXCL5 (LIX, Lipopolysaccharide-induced CXC-chemokine), CXCL9 (MIG, Monokine induced by gamma interferon), CXCL11 (I-TAC, Interferon-inducible T-cell alpha chemoattractant), CXCL13 (BCA-1, B-cell attracting chemokine 1) and CX3CL1 (Neurotactin) in caput epididymidis extracts (**A**) and plasma (**B**) from 6 month-old *wt* and *IDO1−/−* animals. *P≤0.05; **P≤0.01.

The KYN:TRP ratio (KTR) is commonly used as an indicator of the activation of the KYN pathway [15]. As shown in Figure 1A, the KTR in wild-type caput epididymal extracts is about two orders of magnitude higher than in corresponding plasma samples. This confirms that in *wt* animals the mouse caput epididymidis is in a special immune/inflammatory state compared to the systemic compartment. By contrast, in *Ido1^−/−^* animals, the KTR in the caput epididymis does not differ from that in plasma, suggesting that IDO1 is the major provider of KYN in the former tissue. To monitor the inflammatory status of the caput epididymis, we measured the classical pro-inflammatory cytokines TNF-α and INF-γ in both *wt* and *Ido1^−/−^* caput epididymal extracts. As depicted in Figure 1B, in the absence of IDO1 these cytokines were significantly up-regulated in caput epididymal extracts, while plasma levels did not differ significantly between *wt* and *Ido1^−/−^* animals. The up-regulation of these cytokines in the caput epididymidis of *Ido1^−/−^* animals suggests an aggravated inflammatory state. This is supported by the fact that caput epididymidal extracts of *Ido1^−/−^* animals also contained higher levels of the soluble TNF receptors sTNF-RI and sTNF-RII compared to *wt* extracts (Fig. 1C) which are often seen as classical counteractive phenomena to diminish TNF-α-mediated pro-inflammatory signal. The increase in the inflammatory status of the *Ido1^−/−^* caput epididymidis was further confirmed by the observation, that several inflammatory chemokines including CCL3 (MIP-1α), CCL5 (RANTES), CCL11 (Eotaxin-1), CCL24 (Eotaxin-2), CCL25 (TECK), CXCL9 (MIG), CXCL11 (I-TAC), and CX3CL1 (Neurotactin) were also up-regulated in *Ido1^−/−^* caput epididymidal extracts as shown in Figure 2A. The up-regulation of these chemokines was confined to the caput epididymidis, as the corresponding plasma levels were not elevated (Fig. 2B). The specificity of the epididymal response is further attested by the fact that amongst the various chemokines surveyed not all of them were up-regulated. For instance, the concentrations of CCL1 (TCA-3), CCL2 (MCP1), CXCL5 (LIX) and CXCL13 (BCA-1) were not elevated in caput epididymal extracts of *Ido1^−/−^* animals, and two of them, namely CCL2 (MCP1) and CXCL5 (LIX), were found to be even down-regulated in the corresponding plasma samples (Fig. 2A and 2B, right bars).

### Other Indicators of *Ido1^−/−^* Caput Epididymis Inflammatory Status

**Figure 3:**
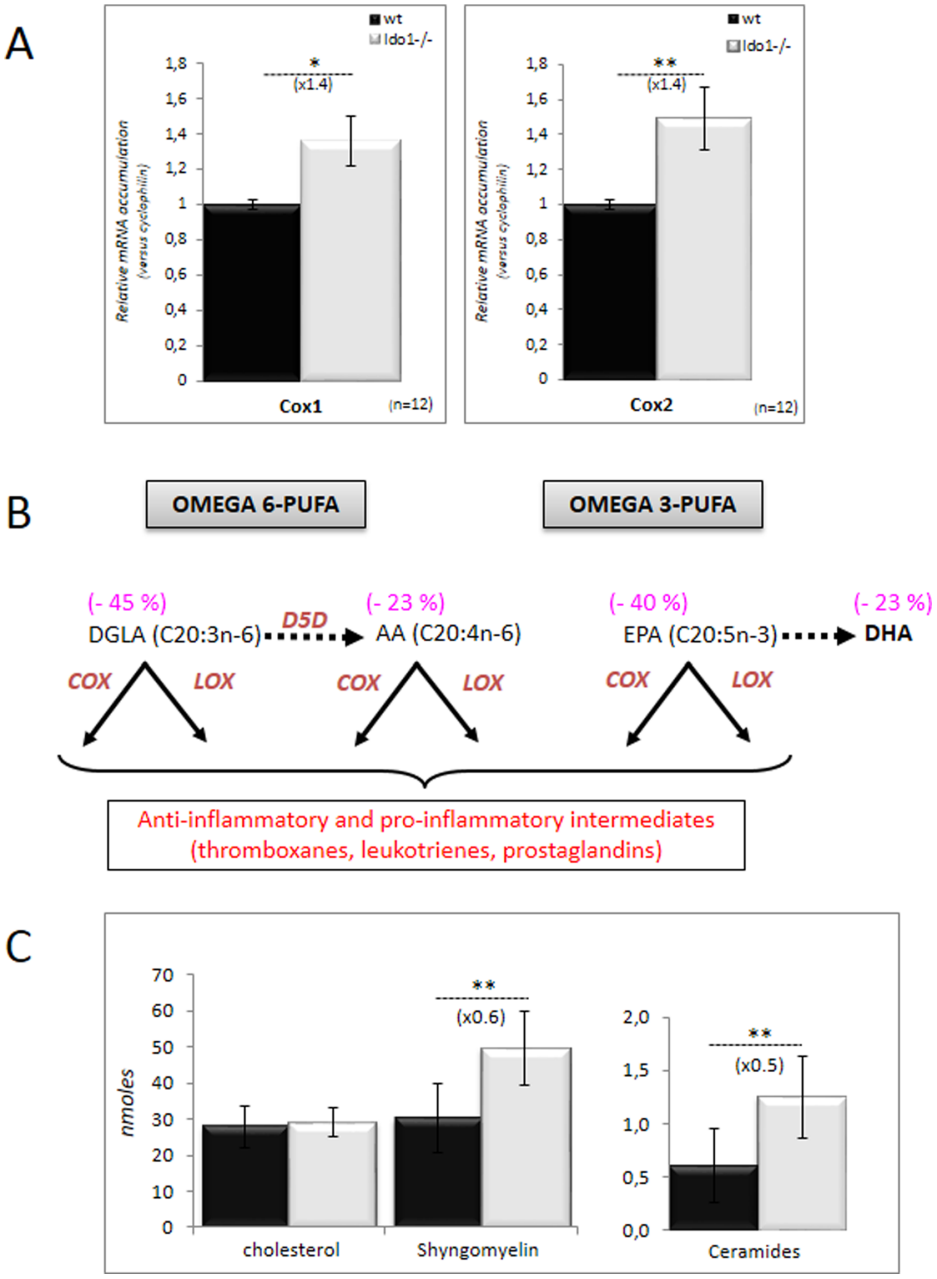
Other inflammatory markers of the IDO1^−/−^ caput epididymidis. **A:** Quantitative RT-PCR estimations of *COX1* and *COX2* mRNA accumulation in caput epididymidis samples from *wt* (black bars) and *IDO1^−/−^* (grey bars) male mice. For quantification of transcripts, the relative method was used to calculate mRNA levels relative to *Cyclophilin B* standard. WT levels were set as 1 in the Y-axis. Mean +/− SEM; n = 12. *P≤0.05; **P≤0.01. **B:** Schematic representation of the omega 6- (ω6) and omega 3-derived (ω3) fatty acid (FA) intermediates (DGLA, Dihomo-gamma-linolenic acid; AA, Arachidonic acid; EPA, Eicosapentaenoic acid and DHA, Docosahexaenoic acid) precursors of the eicosanoids derivatives including prostaglandins, leucotrienes and thromboxanes. Bracketed numbers given above each FA species indicate difference recorded in the representation of these FA in caput epididymidis extracts of *IDO1^−/−^* animals versus *wt* (n = 6). **C:** Histograms illustrate the recorded differences in sphingolipid intermediates (sphingomyelin and ceramides) concentration in caput epididymidis extracts of *wt* (black bars) and *IDO1^−/−^* (grey bars) from 6 month-old animals. Cholesterol level was taken as a reference to show that the transgenic animals do not present a general disruption in their epididymal lipidic profile.

Inflammatory situations are commonly accompanied by an increase in the mobilization of lipid mediators such as eicosanoids that have both anti- and pro-inflammatory actions [16]. During inflammatory processes part of these eicosanoids are the result of cyclooxygenase activity, essentially the inducible (upon inflammation) cyclooxygenase 2 (COX2), which metabolizes arachidonic acid released from membrane phospholipids. In Figure 3A, we show that the accumulation of both COX1 and COX2 transcripts is more pronounced in caput epididymal extracts of *Ido1^−/−^* than in *wt* controls confirming the more pronounced inflammatory situation of the *Ido1^−/−^* caput epididymis. Since arachidonic acid consumption by COX enzymes is classically accompanied by a distortion of the free fatty acid profiles we have looked at the free fatty acid contents in both *Ido1^−/−^* and *wt* caput epididymal extracts. In agreement with the increase in COX expression, concentrations of all the ω6 and ω3 lipidic intermediates (leading to prostaglandins, leukotrienes & thromboxanes) were significantly reduced in *Ido1^−/−^* caput epididymidis extracts compared to *wt* (Fig. 3B). Reflecting classical pro-inflammatory situations we observed a change in the ω6/ω3 ratio in caput epididymidis extracts of *Ido1^−/−^* animals in favor of the ω6 series [17]. In addition, we observed that the SCD (stearoyl-CoA desaturase) ratio (16∶1n-7/16∶0) is 3-fold higher in *Ido1^−/−^* than in *wt* caput epididymidis extracts and that the levels of unsaturated fatty acids were also increased, both parameters constituting classical signs of inflammation [18]. Finally, amongst other lipid intermediates that are known to be associated with inflammation [19], increased levels of sphingolipids/ceramides were also seen in caput epididymal extracts of *Ido1^−/−^* animals when compared to *wt* (Fig. 3C).

### Impact of IDO-deficiency on T Cell-mediated Responses in the Caput Epididymis

**Figure 4:**
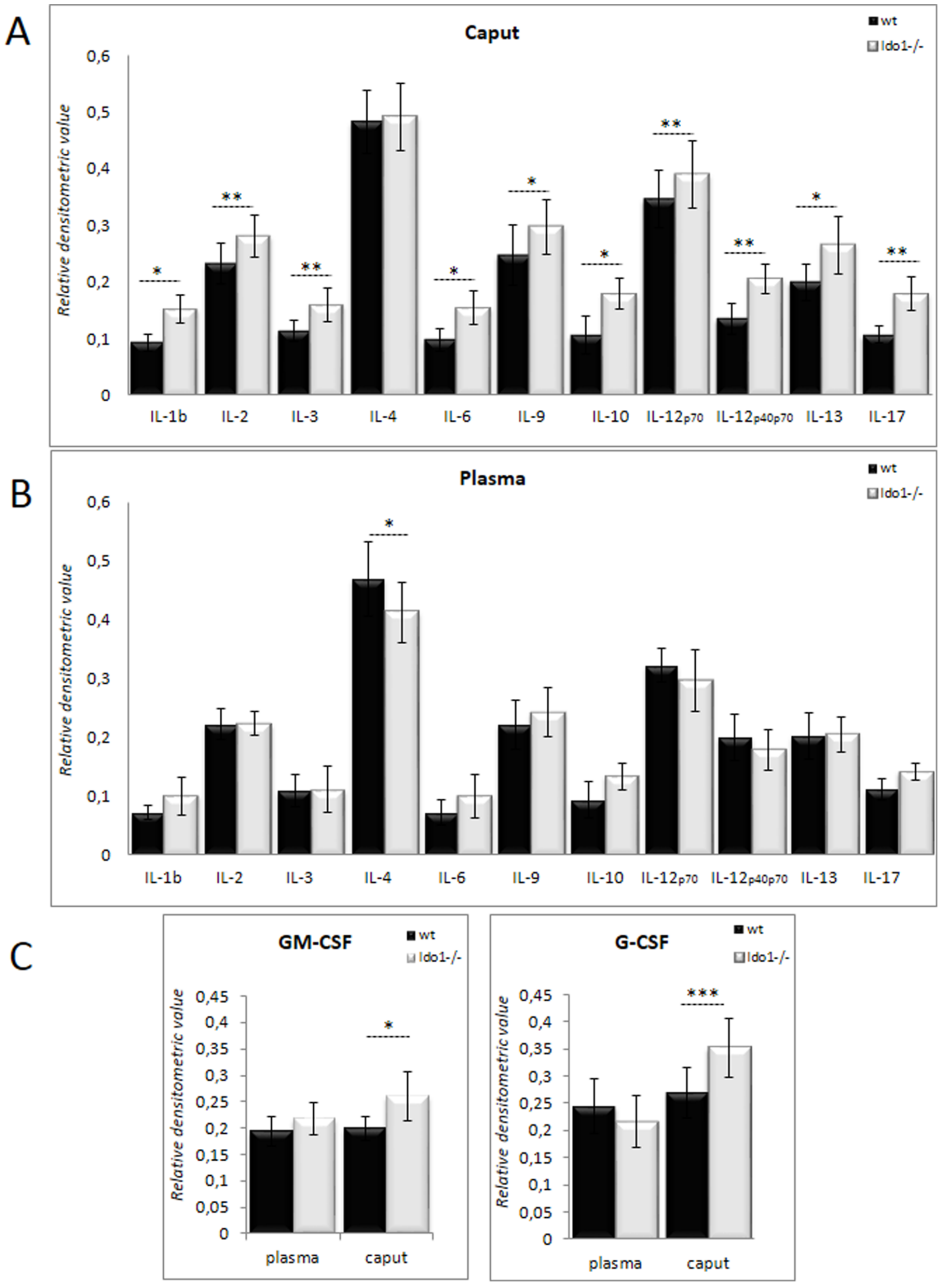
Interleukin profile of the epididymis. Histograms show the levels of the interleukins IL-1ß, IL-2, IL-3, IL-4, IL-6, IL-9, IL-10, IL-12p70, IL-12p40p70, IL-13, and Il-17 in caput epididymidis extracts (**A**) and plasma (**B**) from 6 month-old *wt* and *IDO1−/−* animals. *P≤0.05; **P≤0.01.

Both KYN production and tryptophan depletion resulting from IDO activity are known to specifically control the activation and differentiation of T-cell subpopulations [20]. To verify whether IDO1 deficiency affects T cell-mediated responses in the caput epididymidis, we monitored the expression of various interleukins associated with T-cell differentiation and activation. It is obvious from Figure 4A that, with the exception of IL-4, all other T cell-related cytokines monitored including IL-1ß, IL-2, IL-3, IL-6, IL-9, IL-10, IL-12p70, IL-12p40p70, IL-13, and IL-17 were significantly up-regulated in *Ido1^−/−^* caput epididymal extracts. In plasma, on the other hand, with the single exception of IL-4, no significantly increased lymphokine levels were observed (Fig. 4B). These data suggest that a T-cell response is engaged in the caput epididymidis of *Ido1^−/−^* animals, which is further supported by significantly increased concentrations of GM-CSF and G-CSF in the epididymis of *Ido1^−/−^* animals, two colony-stimulating factors known to be produced by T cells, (Fig. 4C).

### IDO1 Deficiency in the Caput Epididymidis Alters the Th17/Treg/Th1/Th2 Equilibrium

**Figure 5:**
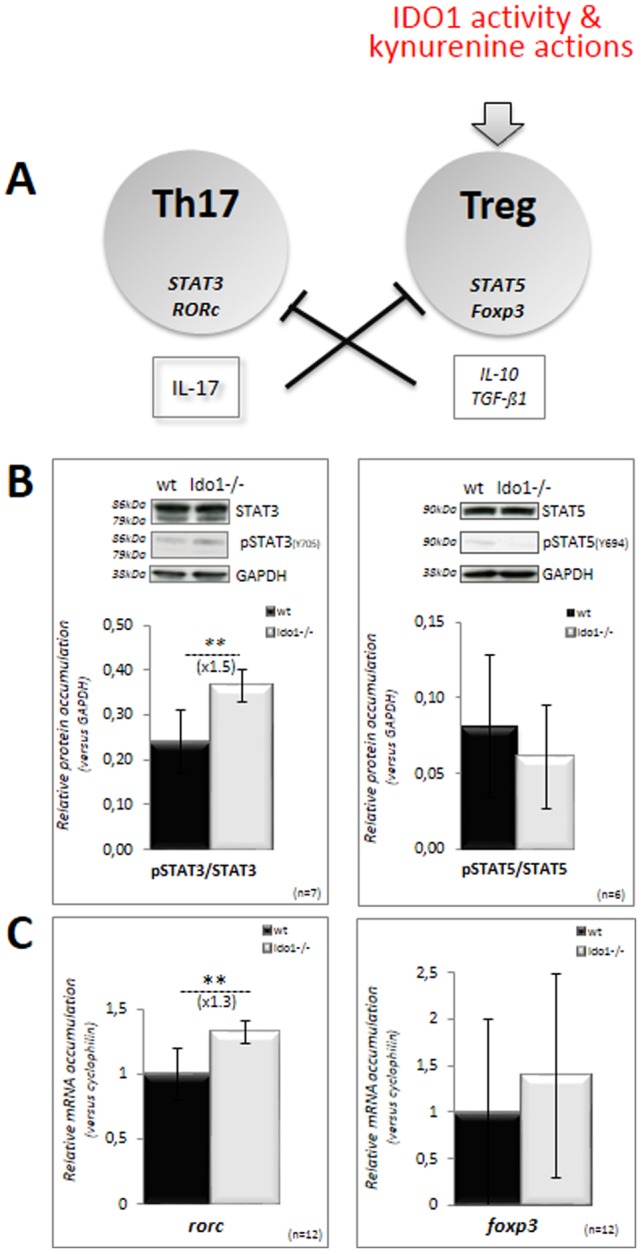
Th17/Treg equilibrium in the caput epididymidis. **A:** Differentiation of the Th17 and Treg lineages from naïve T cells are mutually exclusive *via* their respective cytokines. STAT3/RORc drive the differentiation of Th17 cells, while STAT5/FOXP3 drive the differentiation of Treg. In inflammatory or/and tolerogenic situations, IDO1 activity and the resulting KYNs promotes the differentiation of Treg cells and their immunosuppressive actions. **B:** Representative western blots showing the levels of STAT3 and STAT5 proteins and their phosphorylated counterparts phosphoSTAT3 and phosphoSTAT5 upon activation in caput epididymidis extracts from *wt* and *IDO1^−/−^* mice at 6 months of age. Bar graphs display means ± SEM using GAPDH as an internal standard for quantification. (n = 7 for Stat3, and n = 3 for Stat5; **p<0.01). **C:** Quantitative RT-PCR estimations of *RORc* and *FOXP3* mRNA accumulations in caput epididymidis samples from *wt* (black bars) and *IDO1^−/−^* (grey bars) male mice. For quantification of transcripts, the relative method was used to calculate mRNA relative level to *Cyclophilin B* standard. WT levels were set as 1 in the Y axis. Mean +/− SEM; n = 12. **P≤0.01.

Depletion of tryptophan *via* IDO activity as well as KYN actions have been shown to induce the differentiation of naïve T cells into the Treg lineage [21]. The latter is characterized by its immunosuppressive effect mediated by its ability to secrete immunomodulatory cytokines such as IL-10 and TGFß1 [22]. Both cytokines in return slow down the differentiation of the Th17/Th1/Th2 inflammatory subpopulations [23]. Thus, IDO activity influences the Th17/Treg equilibrium in favor of Tregs, allowing immunosuppressive actions and the creation of a tolerogenic situation (Fig. 5A). To evaluate whether this Th17/Treg equilibrium has been modified in caput epididymal tissue from *Ido1^−/−^* animals we used Western blot and real-time PCR to respectively monitor, the STATn-dependent signal transduction pathways and the accumulation of key transcription factors that specifically govern Th17 and Treg differentiation from naïve T cells. Figures 5B and 5C show significant increases in the phosphorylation status of the STAT3 signal transducer intermediate and in the expression of the Th17-lineage specific transcription factor RORc, respectively, in *Ido1^−/−^* caput tissue extracts as compared to *wt* extracts. In the same samples we observed neither a change in the phosphorylation status of STAT5 nor in the expression of the FOXP3 transcription factor, both of which are specific for the Treg lineage differentiation program. These data suggest that in caput epididymal extracts of *Ido1^−/−^* animals the Th17/Treg equilibrium is altered in favor of the Th17 subpopulation.

**Figure 6:**
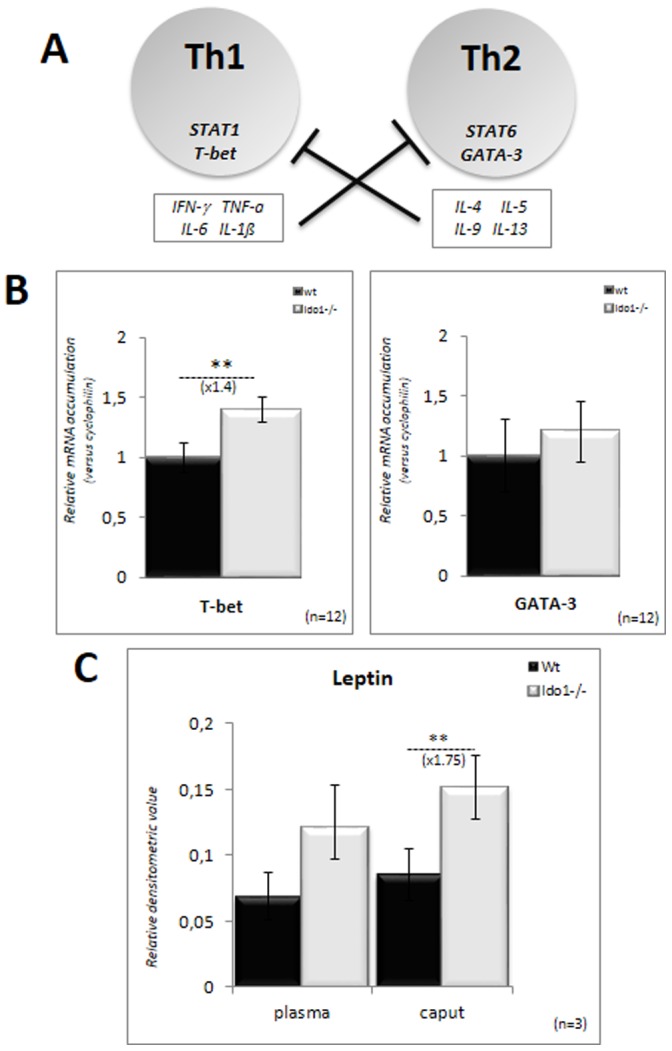
Th1/Th2 equilibrium in the caput epididymidis. **A:** Differentiation of the Th1 and Th2 lineages from naïve T cells are mutually exclusive *via* their respective cytokines. T-bet drives the differentiation of Th1 cells while Gata-3 drives the differentiation of Th2 cells. **B:** Quantitative RT-PCR estimations of T-bet and Gata-3 mRNA accumulation in caput epididymidis samples from *wt* (black bars) and *IDO1^−/−^* (grey bars) male mice. For quantification of transcripts, the relative method was used to calculate mRNA levels relative to *Cyclophilin B* standard. WT levels were set as 1 in the Y-axis. Mean +/− SEM; n = 12. **P≤0.01. **C:** Histograms show the levels of leptin in caput epididymidis extracts and plasma from 6 month-old *wt* and *IDO1−/−* animals. **P≤0.01.

As increased representation of the inflammatory Th17 subpopulation in *Ido1^−/−^* caput epididymis should affect the Th1/Th2 equilibrium [23] we show in Figures 6A-B that the Th1-lineage specific transcription factor T-bet was significantly up-regulated in caput epididymal extracts of *Ido1^−/−^* animals, while expression of the Th2-lineage specific transcription factor GATA-3 did not change. These data support the notion that the caput epididymis of *Ido1^−/−^* animals is engaged in a Th1 response. Consistent with this hypothesis, a significant increase in the leptin content of caput epididymal extracts from *Ido1^−/−^* animals was found, while plasma levels did not differ significantly from those of *wt* animals (Fig. 6C). Leptin is known to prime the generation of pro-inflammatory adaptative Th1 responses after T^CD4+^-cells have encountered antigens following their interaction with antigen presenting cells (APC) [24], [25].

### IDO1 Deficiency does not Alter the Overall Leucocyte Representation in Caput Epididymis

**Figure 7:**
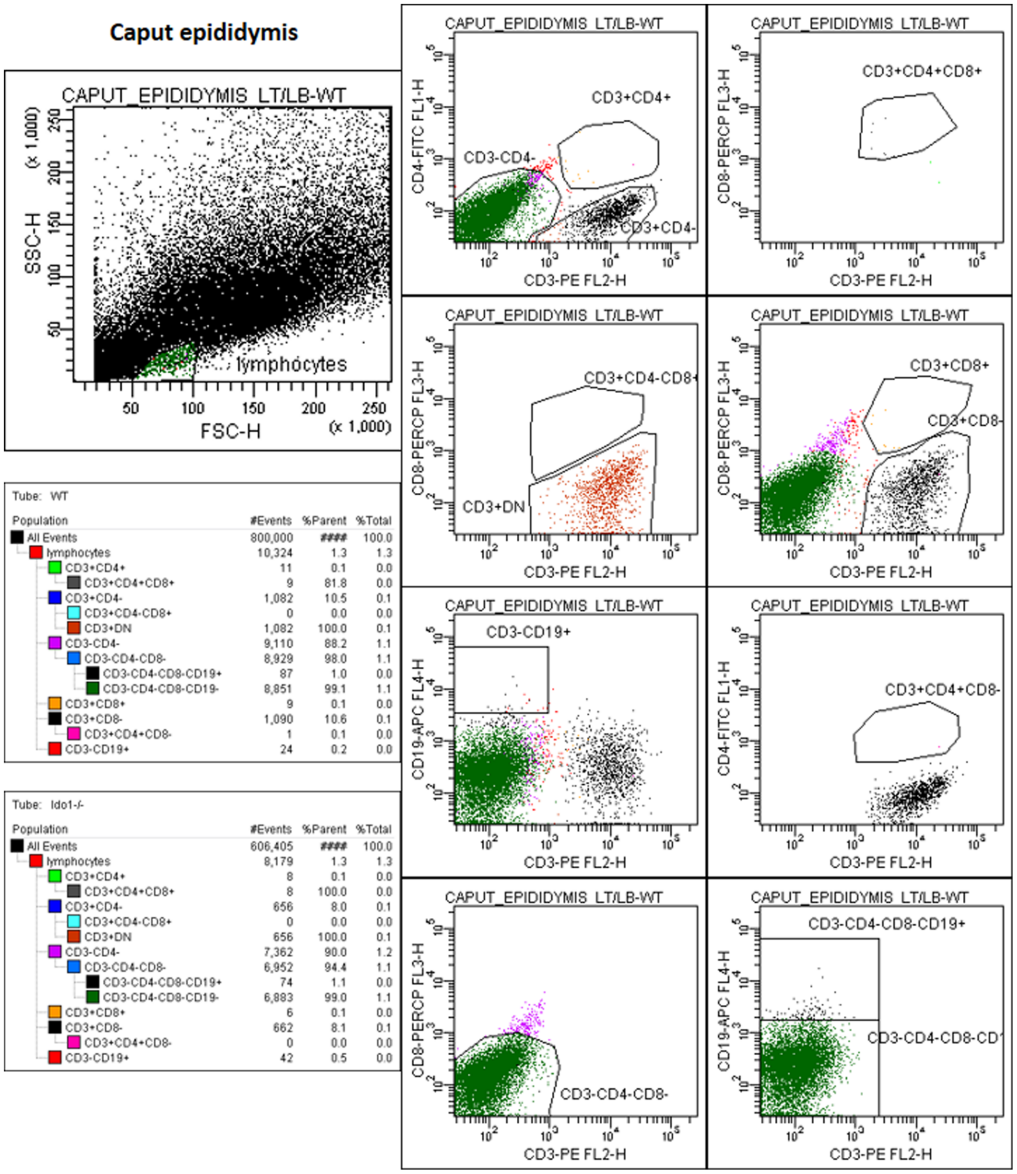
Flow cytometry analysis of lymphocytes populations amongst *wt* and *Ido1^−/−^* caput epididymal cells. Cytogrammes show the gating strategy used to identify the different lymphocytes subpopulations. The lymphocyte gate was defined according to cell size (FSC) and granularity (SSC) criteria. The two tables show the number of cells (events) for each population gate in *wt* (left) and *Ido1^−/−^* (right) animals. They also show the proportion of each population among all acquired cells (% total).

FACS analysis was used to look at the distribution of leucocytes in caput epididymis tissue samples dissociated by collagenase treatment in both *wt* and *Ido1^−/−^* animals. Figure 7 shows cytofluorometric analyses of gated lymphocyte subpopulations in *wt* and *Ido1^−/−^* caput epididymis samples (n = 5). Globally there is no dramatic change in the representation of the various leucocyte lineages that we have been able to monitor in the different issue extracts. In addition, the analysis revealed that B cells and CD3^+^ cells were quite rare in the caput epididymal tissue, whatever the genetic background, and that most of the lymphoid cells were triple negative (CD3^−/^CD4^−/^CD8^−^) cells. Regarding the few CD3^+^ cells, they were essentially found to be of the CD3^+^ double negative type (devoid of CD4 and CD8), the so-called CD3^+DN^ or TDN.

## Discussion

The present data indicate that IDO1 and its downstream metabolites, the KYNs, participate in the establishment of an immunological equilibrium within the caput epididymis of mice. This is supported by our observations that in the absence of IDO1 expression (i.e., in the *Ido1^−/−^* mouse model [8]) an increase in the concentrations of classical inflammatory cytokines (IFN-γ, TNF-α, IL-6, IL-1ß) as well as in various chemokines associated with T cell-driven inflammatory situations was seen, but solely in caput epididymidis extracts and not in plasma. A change in the inflammatory status of the caput epididymidis in *Ido1^−/−^* animals was also indicated by the up-regulation of several T cell-generated lymphokines and the over-expression of the inflammation-induced cyclooxygenase, COX2. As expected, COX2 induction in the epididymis of *Ido1^−/−^* animals was associated with increased consumption of ω3- and ω6-PUFA, which serve as precursors for both pro-inflammatory and anti-inflammatory effectors including prostaglandins, leukotrienes and thromboxanes [16]. In addition, we show here an increase in the sphingolipid/ceramide pathway in caput epididymidal extracts of *Ido1^−/−^* animals (Fig. 3C) that may, in part, explain the activation of COX2, because sphingolipids and particularly ceramides have been shown to actively participate in TNF-α-mediated inflammatory responses (for a recent review see: [19]). It is worth noting that the epididymis is rather unusual in this regard as it is characterized by its constitutive expression of COX2 [26], [27]. In any other tissue, COX2 expression is solely induced by inflammatory stimuli. The constitutive expression of both COX2 and IDO1 reinforces the idea that the caput epididymidis is characterized by the maintenance of a permanent immune-tolerant state. We show here that both COX1 and COX2 expression levels were upregulated in *Ido1^−/−^* caput extracts compared to *wt*. Although it is commonly believed that the constitutive isoform COX1 has little or no involvement in regulating immune responses [28], there are recent reports suggesting that COX1 is actively involved in immunoregulation [29] and that part of its effect is mediated *via* IL-17 production by Th17 cells [30]. Our data are consistent with these statements since we observed a shift in the Th17/Treg equilibrium towards Th17 cells and an associated increase in the concentration of IL-17, in the caput epididymides of *Ido1^−/−^* animals.

Given the recent report that the mouse epididymal duct, and especially the caput region is lined with antigen presenting dendritic cells [31], it is expected that an increased inflammatory situation, as it is the case in *Ido1^−/−^* animals, will exacerbate sperm-antigen presentation in the caput. In this process, antigen presenting cells (APC) and the cytokine environment prime directly and indirectly naïve T cells to differentiate into various cell subtypes known as Th1, Th2, Th17 and Treg. The equilibrium in these various T-cell subpopulations is exquisitely regulated through mutually exclusive cytokine-mediated paracrine regulations with the reciprocal inhibition of Th1 and Th2 on one side and of Th17 and Treg on the other side, as well as the general inhibition of the Treg population over the inflammatory Th17, Th1 and Th2 populations [32]. IDO activity has clearly been reported to be an inducer of the Treg differentiation pathway, thus partly explaining its immunosuppressive actions and the creation of tolerogenic states in situations where IDO is upregulated [33]. It was therefore expected, that lack of IDO1 expression in the caput epididymidis territory would promote the differentiation of the Th17 subpopulation. In the absence of an easy way to appreciate quantitatively the caput epididymal Th17 subpopulation we show here that the factors that promote the Th17 differentiation (ie, STAT3 and RORc) are up-regulated in the *Ido1^−/−^* caput extracts.

### Chemokines and Cytokines Production in Caput Epididymidis of *Ido1^−/−^* Animals Argue for a Th1-driven T Cell Response

Concerning the chemokine/cytokine status, it is interesting to note that amongst the up-regulated chemokines in *Ido1^−/−^* caput epididymal extracts we found CCL3 (MIP-1α) and CCL5 (RANTES). Both promote development of IFN-γ-producing Th1 lymphocytes directly or indirectly by increasing IL-12 production by APCs [34]. Accordingly, IL-12 and IFN-γ levels were also increased in the caput epididymis of *Ido1^−/−^* animals. In addition, levels of IFN-γ-mediated CXC chemokines including CXCL9 (MIG), CXCL11 (ITAC) and CX3CL1 (Neurotactin) were also elevated in the caput epididymides of *Ido1^−/−^* animals. These chemokines are known to recruit Th1 lymphocytes during inflammatory processes [34]–[36]. Taken together, these observations suggest that the chemokine/cytokine environment of the caput epididymidis of *Ido1^−/−^* animals is that of a Th1-based response. This conclusion is reinforced by the observation that amongst the chemokines and cytokines that remained unaltered in the caput epididymides of *Ido1^−/−^* animals were CCL2 (MCP1) and IL-4. The latter is the classical Th2 cytokine, whereas CCL2 is known to inhibit the production of IL-12 and to enhance the production of IL-4 by activated Th2 cells [37]. Our conclusion that lack of IDO1 expression triggers a Th1-driven cellular immune response in the caput epididymidis is also supported by our observations that Th1-specific cytokines (IL-2, TNF-α and IFN-γ) were found at higher levels in caput epididymal extracts of *Ido1^−/−^* than in *wt* animals. Increased leptin levels in caput epididymis extracts of *Ido1^−/−^* animals also support a Th1 response, as leptin has been shown to stimulate the Th1 pro-inflammatory cellular immune response [24], [25]. Leptin has also been shown to play pro-inflammatory and immunomodulatory roles in various autoimmune diseases characterized by Th1 autoreactivity [38], [39]. Finally, concurring with the cytokine/chemokine Th1 profile, the Th1 cell-specific transcription factor, T-bet, is up-regulated in *Ido1^−/−^* caput epididymal extracts. Altogether, these observations support the idea of an inflammatory Th1-mediated cellular immune response in the caput epididymis of *Ido1^−/−^* animals. Promotion of a Th1 response in the epididymis of *Ido1^−/−^* animals is expected in the context of a weaker immune-tolerant environment against sperm antigens that should stimulate a Th1-driven autoimmune reaction [40]. In addition, it has been reported that kynurenines including 3-hydroxy-arachidonic acid (3-OH-AA) and 3-hydroxykyurenine (3OHK) inhibit the actions of Th1 cells and enhances the action of Th2 cells [41]. Other reports have shown that stimulation of IDO activity in dendritic cells diminishes Th1 response [42]. Conversely, it was also reported that 1-methyltryptophan-mediated inhibition of IDO activity enhances Th1-associated inflammation [43]. Therefore, in the absence of IDO1 and its downstream metabolites, as it is the case in the epididymis of the *Ido1^−/−^* animals [7], one should expect a shift of the Th1/Th2 equilibrium towards Th1. This behaviour in agreement with the hypothesis that the immunosuppressive action of IDO and its role in inducing immune tolerance is ensured by its inhibitory effect on the Th1 subpopulation [40].

### Caput Epididymidis of IDO1-deficient Animals Copes with Increased Local Inflammation and Avoids Compromise of its Immune-tolerant Status Towards Spermatozoa

We have shown above that IDO1 deficiency promotes inflammation in the caput epididymidis *via* a shift in both Th17/Treg and Th1/Th2 balances, respectively, in favor of Th17 and Th1 pro-inflammatory T cells. However, this situation does not lead to acute inflammatory conditions such as leucocytospermia and granulomas. This suggests that compensatory mechanisms are at work to maintain the immune balance of the epididymis in *Ido1^−/−^* animals. In confirmation of this *status quo* situation, our preliminary investigations *via* FACS analyses showed that the caput epididymal leucocyte distribution is not modified when *wt* animals were compared to *Ido1^−/−^* (Fig. 7). In agreement with previous reports it also showed that most of the lymphoid cells were triple negative (CD3^−/^CD4^−/^CD8^−^) meaning that they could be either dendritic cells (DC) or of the natural killer (NK) lineage and that B cells and CD3^+^ lymphoid cells were scarce [44]. It is interesting to note that the CD3^+^ cells were found to be mostly of the CD3^+^ double negative type (devoid of CD4 and/or CD8), the so-called CD3^+DN^ or T^DN^ which have been added recently to the increasing list of T cells that exert T regulatory functions similar to those of classical Treg (CD4^+^CD25^+^FoxP3^+^) cells [45]–[49]. Especially, T^DN^ were very recently shown to be involved in the prevention of autoimmune responses engaged in the inflamed epididymis after vasectomy [50].

**Figure 8:**
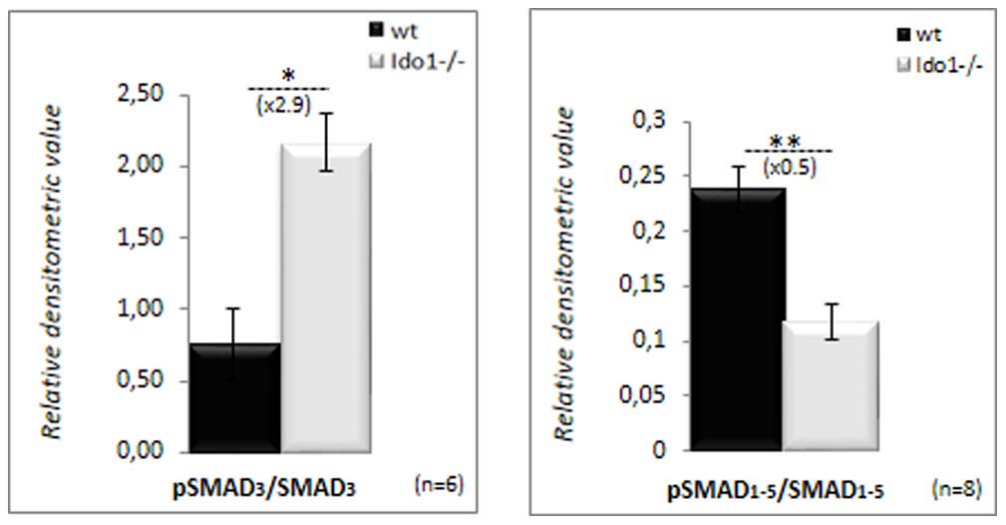
Monitoring of the activation of the TGFß/BMP pathway in caput epididymis extracts. Bar graphs showing the levels of Smad3 and Smad1-5 proteins and their phosphorylated counterparts phosphoSmad3 and phosphoSmad1-5 upon activation in caput epididymidis extracts from *wt* and *IDO1^−/−^* mice at 6 months of age. Bar graphs display means ± SEM using GAPDH as an internal standard for quantification (n = 4 for Smad3, and n = 8 for Smad1-5; *P≤0.05; **p<0.01).

Other non-exclusive hypotheses can be brought forward to explain the new immune equilibrium found in the caput epididymis of the IDO1-deficient animals and the preservation of the tolerogenic environment towards spermatozoa. First, we show here that despite the absence of IDO1 expression and its known inducing effect on the Treg lineage [51], the differentiation of the Treg subpopulation is not dramatically reduced in the epididymis of *Ido1^−/−^* animals. Beside the IDO1/KYN pathway, the immunosuppressive factor TGF-ß1 was shown to act as a Treg autocrine inducer [52]. It is worth noting that *via* comparative gene expression array analyses (not shown) we revealed *Bmp8a* to be among the genes most strongly induced in *Ido1^−/−^* caput epididymides. BMP8a belongs to the TGF-ß family and was shown previously to participate in the differentiation of the caput epididymal epithelium [53], [54]. Interestingly, lack of BMP8a and BMP7 expression in *Bmp8a^−/−^* and *Bmp8a/Bmp7^−/−^* mice provoked inflammatory situations in the caput epididymides of these animals characterized by the occurrence of sperm-mediated granulomas [54]. These observations support the idea that a TGF-ß family member such as BMP8a might participate in the immune control of the adult caput epididymidis. It is thus possible that through an anti-inflammatory autocrine/paracrine signaling cascade involving the TGF-ß/BMP/activin pathway the *Ido1^−/−^* epididymal epithelium partly recovers its immune balance by maintaining the Treg population or by negatively controlling the expansion of the Th17 sub-lineage. This hypothesis is supported in the present study by our preliminary observation that in *Ido1^−/−^* caput epididymidis extracts the SMAD3 intracellular effector of the TGF-ß pathway is activated (Fig. 8). Which cell type(s) in the epididymis epithelium is (are) involved in that phenomenon remains to be clarified. Secondly, the increase in the immunosuppressive cytokine, IL-10, we observed in the *Ido1^−/−^* caput extracts may also participate in the new immune equilibrium. Although Treg is the major provider of IL-10, it has been shown that several other cell types can also produce it; including Th1, Th2, cytotoxic T cells, B lymphocytes, mast cells, mononuclear phagocytes, APCs as well as T^DN^ cells [34], [55]. More investigations will be necessary to identify which cell(s) type(s) in the caput epididymis of *Ido1^−/−^* animals is responsible for the increased production of IL-10. Thirdly, the earlier reported 50% decrease in proteasomal activity in *Ido1^−/−^* caput extracts [7] may constitute yet another regulatory anti-inflammatory process to diminish both the production of inflammatory cytokines and, MHC-mediated antigen presentation in chronic inflammatory situations as it has been suggested elsewhere [56], [57].

In conclusion, we have shown here that IDO1 plays a role in maintaining the unusual immune balance that characterizes the caput epididymidis. In the absence of IDO1 expression, local inflammation is promoted by the engagement of a Th1-driven T-cell response as expected when autoimmunity is stimulated. Despite the absence of this immunosuppressive player the epididymis of the *Ido1^−/−^* mice copes with the pro-inflammatory situation and maintains immune-tolerance. Our data suggest that TGF-ß signaling, IL-10 up-regulation and impairment of caput epididymis proteasomal activity may represent means by which the epididymis of *Ido1^−/−^* animals restores its immune balance.
